# The Mental Health of Older Adults in the Densely Populated Areas of Tacna Region—Peru, 2021: Implications of the COVID-19 Information

**DOI:** 10.3390/ijerph191811470

**Published:** 2022-09-12

**Authors:** Elena Cachicatari-Vargas, Karimen Jetzabel Mutter Cuellar, Wender Florencio Condori Chipana, Flor de Maria Miranda Socasaire, Ángel Acevedo-Duque, Orfelina Arpasi-Quispe

**Affiliations:** 1 Faculty of Health Sciences, Universidad Nacional Jorge Basadre Grohmann, Tacna 23001, Peru; 2 Programa de Doctorado en Ciencias Sociales, Universidad Autónoma de Chile, Santiago 7500912, Chile; 3 Graduate School, Norbert Wiener University, Lima 15046, Peru; 4 Faculty of Health Sciences, Universidad Peruana Unión, Lima 15046, Peru

**Keywords:** exposition, COVID-19, mental health, older adults

## Abstract

The purpose of this research was to analyze the implications of exposure to various news channels that broadcast information on COVID-19 and their impact on the mental health of older adults in the sparsely populated area of the Tacna Region during the year 2021. The present study used a descriptive correlational type of quantitative approach on a sample of 389 older adults aged 60 years and over, who were recruited by non-probabilistic convenience sampling. For the application of the survey technique, the instrument used was a questionnaire modified by the authors. In terms of research ethics for the development of the study, the respondents provided informed consent, and other ethical considerations were addressed. In relation to sociodemographic variables of mental health, it was found that women had a greater incidence of anxiety (*p* < 0.01) and that people with fewer years of study had a greater incidence of depression (*p* < 0.01) and anxiety in sparsely populated areas. Exposure to news through television was associated with depression, and news obtained from other people was associated with depression (*p* < 0.001). An association was also found between the number of hours of television news and stress (*p* < 0.05), and radio news was associated with anxiety (*p* < 0.05). In terms of psychological consequences, the highest mean for television exposure was fear, while the greatest psychological consequence of radio news was fear, followed by stress and awareness. Finally, negative, inverse, and significant relationships were found that indicate protective factors, such as depression with awareness and indignation, and anxiety was inversely related to awareness.

## 1. Introduction

A second entry point that would allow greater visibility of older people in the 2030 Agenda is the document’s insistence on the need for data to be disaggregated, among other factors, by age [1]. The 2030 Agenda conceptualizes the establishment of indicators and disaggregation of data as mechanisms for monitoring progress in the implementation of the SDGs [2]. Among several mentions of the need for disaggregated data, under the heading “Data, Monitoring and Accountability”, a specific target (17.18) aims to “enhance capacity-building support to developing countries to significantly increase the availability of timely, reliable and high-quality age-disaggregated data”, among other factors mentioned.

This inclusion is vitally important, given the frequent absence of post-pandemic data on people aged 60 years and older and their health, which are simply ignored by many statistical systems and administrative records [3,4]. Improving the capacity of States to produce age- and health-disaggregated data would also enable better identification of groups left behind and the analysis of intersectional or overlapping discriminations or vulnerabilities.

The motto and cardinal principle of the 2030 Agenda is precisely “no one is left behind” [5]. This implies that each country must identify, in its own context, those groups that have been left behind in traditional human rights terms who have suffered discrimination and marginalization and do not fully enjoy their rights. This is relevant because, in 2020, there was an unexpected health situation that we are still facing today: the COVID-19 pandemic, declared as such on 11 March 2020 by the Director-General of the World Health Organization (WHO), Tedros Adhanom Ghebreyesus [6].

In Peru, the first imported case of COVID-19 was confirmed on 5 March 2020 in a person with a history of travel to Spain, France, and the Czech Republic [6]. Therefore, by Supreme Decree No. 044-2020-PCM published on Sunday, 15 March 2020, in the official gazette El Peruano, the government declared a state of national emergency due to the serious circumstances affecting the life of the nation as a result of the COVID-19 outbreak [7]. The government adopted measures of mandatory social isolation (quarantine), restricting the exercise of constitutional rights related to personal freedom and security, inviolability of the home, freedom of assembly and transit in the territory, and access to essential services and goods, with restrictions on commercial activity, cultural activities, establishments, recreational activities, and hotels and restaurants, as well as the temporary closure of borders and reduction in transportation supply.

This leads us to reflect that, in Peru, there are large age groups: 64.9% (6,493,809) of the inhabitants of Lima are between 15 and 59 years of age, 18.5% (1,850,730) are between 0 and 14 years of age, and 16.6% (1,659,602) are over 59 years of age. People over 60 years of age are considered the most vulnerable to the coronavirus (COVID-19), especially those suffering from chronic diseases (diabetes, arterial hypertension, cancer, and cardiovascular diseases, among others). 

The population of older adults is constantly growing [7], which generates serious challenges for medical and social care systems. Older people deserve not only a longer life expectancy but also a satisfactory quality of life during the final stage of life [7]. The pandemic situation has revealed that older patients are more prone to SARS-CoV-2 infection, a severe disease course and death, which is associated with pre-existing comorbidity. 

In this sense, the aim of this research was to analyze the implications of exposure to various news channels that broadcast information on COVID-19 and their impact on the mental health of older adults in the Tacna Region during the year 2021. The paper is structured as follows: the introduction is followed by a review of the literature framing the study and the purpose of the work. Then, the methodology is described: the present study used a descriptive correlational type of quantitative approach with the application of the survey technique; the instrument was a questionnaire modified by the authors. From the ethical point of view of the investigation, the respondents provided informed consent, and other ethical considerations that are mentioned in the considerations section were addressed. The results are then discussed, demonstrating the importance of the implications of exposure to different news channels that disseminate information about COVID-19 and their impact on the mental health of older adults in the Tacna Region during the year 2021. 

## 2. Background

### 2.1. Emotional Manifestations in the Elderly during the Pandemic

The COVID-19 pandemic, an unexpected, unusual, unthinkable, and surprising event, has become a stress factor with impacts on all levels of life: on society as a whole and its organizations, on political, socioeconomic, and health management, and on the mental and physical health of individual healthy people, with a focus on those most likely to be affected, given that the pandemic has had a disproportionate impact on vulnerable people [8,9]. Given the current situation caused by COVID-19, the rapid spread between countries, and the health effects of this virus, one of the international public health strategies to curb its spread has been containment. People are kept isolated and sheltered in their homes, which implies a drastic change in daily life activities and behaviors.

**Figure 1:**
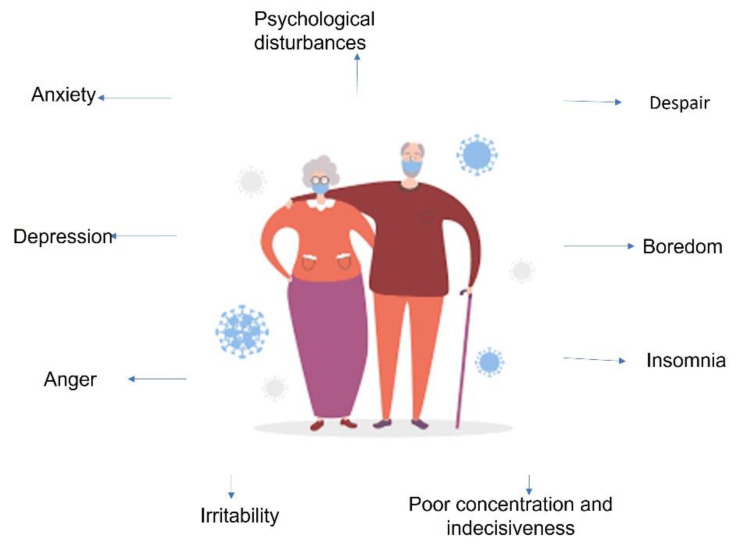
Emotionality of older adults in a sparsely populated area in a pandemic.

Studies in previous epidemics have revealed a wide and profound range of psychosocial consequences at the individual and community levels during outbreaks [10]. Social isolation, restricted mobility, and limited contact with others have increased people’s vulnerability to multiple psychological disorders, despair, boredom, insomnia, lack of concentration and indecisiveness, irritability, anger, depression, anxiety, distress at not having physical contact or contact with family and friends, and not being able to develop a normal life routine (see Figure 1). 

The emotional manifestations mentioned above were observed in the city of Tacna during the first wave of the pandemic, which lasted from July to September 2020, as well as during the second wave, which began in January 2021 and is rising significantly, showing an exponential death growth rate of 68% among the population, with the male population of adults over 60 years of age being the most affected, according to statistics issued by the National Centre for the Estimation, Prevention and Reduction of Disaster Risk (CENEPRES) [11].

These psychological disorders can range from isolated symptoms to the development of a mental disorder [11,12]. The over-60 age group appears to be more vulnerable to becoming seriously ill from the virus and has a higher probability of death, which is associated with its higher prevalence of frailty and vulnerability to adverse events, disability, and dependence related to decreased physiological reserve, advanced age, and pre-existing medical conditions. Previous comorbidities or chronic diseases (heart disease, COPD, asthma, hypertension, diabetes, obesity, and chronic renal failure), which are present in our older adult population, are important risk factors for the vulnerability to and severity of COVID-19 disease [13,14].

### 2.2. Impact of COVID-19 Information on Older Adults

According to [15,16], there is a term derived from the conjunction between the words epidemic and information, determined as “infodemic”, referring to the large volume of information on a subject, which can increase exponentially in a short time for a given incident, such as the coronavirus pandemic (COVID-19), including scientific and technical information, with the possibility of presenting incorrect data and false news that could harm people’s discernment.

According to [17,18], this situation generated an interest among the population in obtaining information about the pandemic, with people constantly exposed to news about risks that deepened feelings of concern and personal vulnerability. According to [19,20], part of the information disseminated by the media was that the highest mortality from COVID-19 occurred in people over 60 years of age. Therefore, age and comorbidities, such as non-communicable diseases, arterial hypertension, cardiovascular diseases, obesity, and diabetes mellitus, among others, were considered risk factors for contracting COVID-19. According to the Technical Documentation of the Peruvian Ministry of Health [21], an older adult is considered to be an individual aged 60 years or older. In this sense, the same institution in Europe explored the discourses, themes, and representations of old age and the elderly presented in the press during the COVID-19 pandemic in Spain [22]. 

These authors classified the content analysis of the headlines into three categories: favorable, unfavorable, and ambivalent representation, with the results showing that 71.4% of the headlines indicated a negative representation of the elderly [21]. These headlines were divided into subcategories that pointed to difficult living conditions, care in institutions, and a lack of human and material resources, including health and safety supplies. Another subcategory included information about deceased elderly, the third subcategory included headlines that pointed to some vulnerability or characterized the elderly as particularly vulnerable or as victims of crime, and the last subcategory showed the elderly as protagonists of misdemeanors, crimes, or reprehensible activities [21]. A negative connotation exposed in communication media was observed.

The authors of [22,23] indicated that the current pandemic is a new form of stress or trauma, both for the population and for health professionals, generating concerns of generalized panic and anxiety. Such concerns may exacerbate and aggravate symptoms of anxiety and depression in people who are more vulnerable or have a diagnosis of mental illness. They identified that news of the death toll, accelerating numbers of new cases, and expansive media attention can increase people’s fears, frustrations, helplessness, and anxiety about the situation, which has a psychological impact on the most vulnerable groups, including older adults [23].

In [24,25], mental health is defined as the “state of well-being in which the individual is aware of his or her own capabilities, can cope with the normal stresses of life, can work productively and fruitfully, and is able to make a contribution to his or her community”, further recognizing that poor mental health is associated with rapid social change, as presented by the COVID-19 pandemic.

Our work and the works of authors in this field, such as [26,27,28], indicate that three weeks after the declaration of the health emergency in Peru due to COVID-19, the will to live and satisfaction with health decreased significantly in the older adult population under study. On the other hand, anxiety increased significantly during the same period and with a large effect size.

## 3. Materials and Methods

The population consisted of 35,156 adults over 60 years of age, according to the National Census 2017: XII Population, VII Housing and III Indigenous Communities of the National Institute of Statistics and Informatics (INE) [29]. The sample was obtained using non-probabilistic convenience sampling and was not determined randomly but according to the availability of people willing to be part of the study sample. For this, the formula of finite samples in sparsely populated areas of Tacna was used. 

The sample size was calculated using the proportion formula: n = N.Z^2^.p. (1 − p) /Z^2^.p. (1 − p). + (N − 1), where “n” is the calculated sample, “N” is the population, “Z” is the standard normalized variable associated with the confidence level, “p” is the actual probability of the event (P = (1 − P) = 0.5, maximum variance assumption), and “e” the sampling error; using a sampling error of 5% and a confidence level of 95%, the result is 383 cases. However, 389 older adults aged 60 years and over from sparsely populated areas of the Tacna Region were considered. An infomediary questionnaire—COVID-19 and its recovery in the mental health of older adults, developed by the Nursing Network in Health of Older Adults of Peru and modified by the authors—was applied; in relation to the ethics of the investigation for the development of the study, the respondents provided informed consent in accordance with the regulation of the General Law of Health on investigations for health.

Prior to data collection, a pilot test of the global instrument used in the present research was applied to 60 older adults aged 60 years or more, taking into account that the population had similar characteristics to the population under study. Cronbach’s alpha coefficient (internal consistency index) obtained a value of 0.851, equivalent to 85.1% reliability for a total of 120 items, considering that the effect of research size is higher than 30%. On the other hand, the results of the validation of the instruments based on Cronbach’s alpha coefficient are presented in detail for each variable, obtaining a result of 0.929 for the Geriatric Anxiety Inventory (GAI), 0.802 for the Geriatric Depression Scale, and 0.806 for the Perceived Stress Scale. Intervention studies involving animals or humans, and other studies requiring ethical approval, should list the authority that provided the approval and the relevant code of ethical approval.


**Variable**

**Alpha**

**Items**
GAI0.929Geriatric Anxiety InventoryGD0.802Geriatric Depression ScaleSP0.806The Perceived Stress Scale

The instrument used was validated through the judgments of experts, physicians, psychologists, and public health specialists so that it could be applied to the study population:

- Sociodemographic profile: For this purpose, we considered sex (male and female), age (years), schooling, marital status (with and without a partner), number of children, type of housing, area of residence, use of health services, and with whom they live, among others.
- The Perceived Stress Scale: This 14-item scale assesses the degree to which people perceive life as unpredictable, uncontrollable, or overloaded. It is a scale of general questions relatively free of specific content for any particular population [30]. Regarding the reliability and construct validity of the Perceived Stress Scale, an adequate level of reliability was determined (α = 0.86), and the measurement model presented an adequate fit: GFI = 0.91, RMSEA = 0.056, NFI = 0.97, CFI = 0.98, and IFI = 0.98 [31].
- Geriatric Depression Scale (GDS): This scale consists of fifteen items with dichotomous answers (yes or no), consisting of positive and negative questions that assess cognitive depressive symptoms. The scale has 15 dichotomous questions with a score from 0 to 15, with a cutoff point of greater than or equal to 5 points being considered indicative of depressive symptoms [32]. This research sought to standardize the Yesavage Depression Scale (reduced version) in non-institutionalized older adults belonging to Day Care Centers in the city of Cali. Of a sample of 500 older adults, 416 were women and 84 were men between 60 and 96 years of age. This methodological type is part of the validation modality of a test. For the standardization of the scale, measures of central tendency and point biserial correlation coefficients were used for each item. The reliability coefficient of the scale is 0.7268, indicating that the GDS-15 scale is highly reliable; 14 of the 15 items are statistically moderately predictive of the depression construct; however, from the psychological analysis, all 15 items are relevant for assessing depressive traits [33].
- Geriatric Anxiety Inventory (GAI): This is a dimensional scale that adapts well and measures three dimensions: cognitive, arousal-related, and somatic symptoms; it has no cutoff point, and the higher the score, the higher the anxiety [30]. Three factors were obtained that explain 50.11% of the variance. The internal consistency obtained for the total scale was 0.91, with alphas ranging from 0.71 to 0.89 for the factors. Significant associations were found between all GAI factors, GAI total score, and depression, rumination, and experiential avoidance (all *p* < 0.01). Women reported higher scores than men for both the GAI total score and all subscales. However, no significant gender differences were found among those with scores above the GAI cutoff score [34].

For data collection, the respective administrative coordination procedures were carried out in order to obtain the corresponding permit. The data collection process was carried out from 22 April to 25 May 2021 among 389 older adults aged 60 years and over in the Tacna Region. The instrument was applied virtually with an approximate duration of 30 min per person.

Once the data were obtained, the instruments were reviewed, ordered, and coded, and a database was created using Microsoft Windows 2017 Excel; then, statistical processing was performed using Statistical Package for the Social Sciences (SPSS) version 25.0 (IBM, Armonk, NY, USA).

After applying the instruments, the data were digitized and processed in the statistical software SPSS v25. Absolute frequencies, percentage frequencies, and summary measures such as mean, standard deviation (SD), skewness, and kurtosis were obtained. Before performing the inferential tests, the normality test was performed, with which it was decided to use non-parametric tests such as the Mann–Whitney U test for differences between two independent samples and the Kruskal–Wallis test for differences between more than two samples. Finally, for relationships between variables, Spearman’s Rho correlation coefficient was used.

## 4. Results

Table 1 shows the implications of sociodemographic variables on mental health. Of the respondents, 56% were women, 85% were between 60 and 79 years old, 59% had a partner, 73% had between 1 and 5 children, and only 3% had no children. Regarding educational level, 36% had secondary education, 30% had primary education, and 24% had higher education; 84% lived in urban areas, and 59% lived with three or more people. Regarding the relationship between sociodemographic variables and mental health, it was found that women had a greater incidence of anxiety (*p* < 0.01), and people with fewer years of education had a greater incidence of depression (*p* < 0.01) and anxiety (*p* < 0.05) (see Table 1).

It was found that women felt more anxiety than men. It was also found that those with fewer years of study felt more depression and anxiety. In Table 2, exposure to co-COVID-19-related news and the association with mental health is presented. The results show that 63% had no exposure to social networks, 35% rarely used television, 33% sometimes listened to the radio, 26% listened to the radio frequently, 38% were rarely exposed to people, and 54% had no exposure to print media. A relationship between exposure to COVID-19-related news and mental health was also found, where it was found that news on television was associated with depression (*p* < 0.01), and news from other people was associated with depression (*p* < 0.001) (see Table 2).

Table 3 presents the number of hours of exposure to news related to COVID-19 and the association with mental health. Here, 62% had no exposure to social networks, 74% were informed by television for 1 to 5 h, 65% were informed by radio for 1 to 5 h, 70% were informed by individuals for 1 to 5 h, and 61% were not informed by the written press. An association was found between the number of hours of television information and stress (*p* < 0.05), and radio information was associated with anxiety (*p* < 0.05) (see Table 3).

Table 4 presents the relationship between psychological consequences and mental health through exposure to television news. Positive, direct, and significant relationships were found for stress with anxiety, fear, and stress; depression with fear; and anxiety with anxiety, fear, and stress, which, having a direct relationship, are risk factors. On the other hand, negative, inverse, and significant relationships were found, which would indicate protective factors, such as stress with indignation, as well as depression with awareness, indignation, and nothing. Finally, anxiety was inversely related to conscientiousness and nothing. Secondly, a relationship between psychological consequences and mental health was found through exposure to radio news. 

Positive, direct, and significant relationships were found for stress with stress and indignation; depression with fear; and anxiety with anxiety, fear, and stress, which, having a direct relationship, are risk factors. On the other hand, negative, inverse, and significant relationships were found, which would indicate protective factors, such as stress with nothing, as well as depression with consciousness and indignation. Anxiety was inversely related to conscientiousness. Finally, a relationship between psychological consequences and mental health was also found for exposure to news through other people. Positive, direct, and significant relationships were found for stress with stress and indignation; depression with anxiety and non-use; and anxiety with anxiety, fear, stress, and security, which, having a direct relationship, are risk factors. On the other hand, negative, inverse, and significant relationships were found, which would indicate protective factors, such as depression with conscientiousness and indignation. Finally, anxiety was inversely related to conscientiousness (see Table 4).

## 5. Discussion 

In December 2019, SARS-CoV-2, a new type of coronavirus that causes the infectious disease COVID-19, was detected in Wuhan, a city in the People’s Republic of China. While most cases are mild, in others, the disease can be severe, causing respiratory distress, pneumonia, kidney failure, and other medical conditions, including death [29]. In January 2020, the World Health Organization (WHO) declared the COVID-19 outbreak a public health emergency of international concern, and on 11 March 2020, it declared it a global pandemic. The world was informed of its high potential for international spread, and warnings were issued about the consequences and impacts on the public health, social, and economic sectors of nations [8,9,10,11,12,13,14,15,16,17,18,19,20,21,22,23,24,25,26,27,28,29,30,31,32,33,34,35]. 

In the current case of the sparsely populated areas of the Peruvian region of Tacna, older adults are the group with the highest COVID-19 mortality rate, making them more vulnerable to stress. In similar research from countries such as Cuba, isolation has caused people aged 60 and over to abandon daily activities and focus only on those they can do at home [30,31,32,33,34,35,36]. Those who were still working stopped working, did not attend grandparents’ circles, the University of the Third Age, or their religious practices, did not visit their friends, relatives, or neighbors, and could not make purchases of any kind; all of these factors could explain the unsatisfactory emotional states identified in the elderly people studied, so most remained on their phones, browsing their social networks [31].

In reference to the present research and, being more specific, according to the data obtained in Table 1, it can be observed that the implications of sociodemographic variables on mental health are that 56% are women, 85% are between 60 and 79 years old, 73% have 1 to 5 children, 36% have secondary education, 30% have primary education, and only 24% have higher education. In addition, 84% live in the city, and 59% live with three or more children. The relationship between sociodemographic variables and mental health shows that women have a higher incidence of anxiety, and people with fewer years of education have a higher incidence of depression and anxiety. This is concordant with studies conducted in Madrid (2020) on the health of the elderly during the pandemic; there was a high degree of mortality in older adults of advanced age, and economic situation was associated with diseases and loneliness [7,25,37,38].

According to the data obtained in the present investigation in Table 2 (exposure to news related to COVID-19 on mental health), 63% have no exposure to social networks, 35% rarely use television, 33% sometimes listen to the radio, 26% listen to the radio frequently, 38% are rarely exposed to news from other people, and 54% have no exposure to print media. A relationship between exposure to news related to COVID-19 and mental health was also found, where news on television was found to be associated with depression (*p* < 0.01), and news through people was associated with depression (*p* < 0.001). It is of great importance to point out that the implementation of new technologies in our current society has important advantages for older people, but they feel distanced from them.

In reference to the above, authors such as [10,15,39] have shown that mental health and its sociocultural environment allow the normal stress of life and ensure participation in productive work life, and the pandemic limited these activities to a greater extent in adults; the pandemic forced preventive isolation, causing emotional effects in this population vulnerable to COVID-19, with weak protective systems. The WHO stated that the effects of the pandemic on mental health are worrying and include anxiety and fear, among others [40,41,42,43].

For the purposes of the results of this research, it is important to highlight the data on surveyed older adults in Table 3, which presents the number of hours of exposure to news related to COVID-19 and the association with mental health. According to the results, 62% have no exposure to social networks, 74% are informed through television for 1 to 5 h, 65% listen to the radio for 1 to 5 h, 70% obtain news through people for 1 to 5 h, and 61% are not informed by the written press. An association was found between the number of hours of television information and stress (*p* < 0.05), and radio information was associated with anxiety (*p* < 0.05).

During confinement, the two factors that most affect the physical and psychological well-being of older adults are loss of habits and routines and psychosocial stress, according to the first study analyzing the psychological impact of COVID-19 quarantine in China [44,45,46]. The alteration of habits during confinement and the establishment of unhealthy habits (e.g., poor eating habits, irregular sleep patterns, sedentary lifestyle, and increased use of screens) can lead to physical problems. 

Authors such as [45,46,47] stated that it is not only health conditions and aging that put older people at risk. Loneliness as an emotion and isolation as a structural condition, in which many of them live, play important roles in their ability to respond to diseases, especially one as contagious and severe as COVID-19 [38,48,49].

Table 4 presents the relationships between psychological consequences and mental health through exposure to television news. Positive, direct, and significant relationships were found for stress with anxiety, fear, and stress; depression with fear; and anxiety with anxiety, fear, and stress, which, having a direct relationship, are risk factors. On the other hand, negative, inverse, and significant relationships were found, which would indicate protective factors, such as stress with indignation, as well as depression with awareness, indignation, and nothing. Finally, anxiety was inversely related to conscientiousness and nothing. Secondly, a relationship between psychological consequences and mental health was found through exposure to radio news [39,40,46].

Finally, a relationship between the psychological consequences and the mental health of older adults through exposure to news through people was found. Positive, direct, and significant relationships were found for stress with stress and anger; depression with anxiety and non-use; and anxiety with anxiety, fear, stress, and security, which, having a direct relationship, are risk factors.

## 6. Conclusions

Regarding the information received in this stage of the investigation, it is evident in the table of sociodemographic variables that 84% are women between 60 and 79 years old, 59% have a partner, 73% have 1 to 3 children, 36% have secondary education, 84% live in urban areas, and 59% live with 3 or more children. It is necessary to have a panoramic view of the demographic characteristics in the Tacna Region, and it can be seen that they are adults with a moderately high educational level who are in the city and close to information and also to the infodemic.

On the other hand, negative, inverse, and significant relationships were found, which would indicate protective factors, such as depression with conscience and indignation. Anxiety was inversely related to conscientiousness. In this study, older adults were targeted by the COVID-19 pandemic, both biologically and psychologically, with conditions such as stress, anxiety, and depression; direct influencing factors were fear of death, fear of relatives, and inability to participate in social and recreational activities.

For the question about elements of exposure to news related to COVID-19 for access to mental health information, it was found that 63% have no exposure to social networks, and 35% rarely have access to television, but the relationship between news exposure and mental health is associated with depression, and news through people is associated with depression. The pandemic has caused the population to be constantly exposed to news that affects discomfort and vulnerability; this situation generated the interest of the population in obtaining information about the pandemic, resulting in constant exposure to news about risks that deepened feelings of discomfort and personal vulnerability.

On the other hand, the information disseminated by the media, particularly statistical reports indicating that the highest mortality from COVID-19 occurred in people over 60 years of age, generated greater fear in this population that is vulnerable to the pandemic. Therefore, age and comorbidities, such as non-communicable diseases, high blood pressure, cardiovascular diseases, obesity, and diabetes mellitus, among others, were considered risk factors for contracting COVID-19. The Peruvian Ministry of Health also considers older adults over 60 to be at risk, which is why we have been able to verify the discomfort that adults feel in the face of this pandemic. We can also say that there is an association between the number of hours exposed to information on television and stress, and information on the radio is associated with anxiety.

### Limitations

From the lived experiences of older adults in this research, we are able to describe that older adults constantly experience stress, anxiety, fear, sadness, and loneliness. This allows us to recognize some important gaps for further research; other significant factors are mental health disorders such as anxiety and depression, which can be exacerbated by the uncertainty of COVID-19.

There were some limitations that arose in the research, such as the health complications presented by older adults in these areas of the Tacna Region, such as coughing, which leads to an increase in respiratory problems, and being able to establish clear information. There were also situations in which many of them showed sadness, uncertainty, and anxiety, symptoms of the information that they had been presented since 2020, and there is uncertainty about the culmination of the pandemic. It is important to converse with and understand each other to reduce stress and fear, including fear of COVID-19, because thinking about COVID-19 can make us sick, so it is important to be happy, think positive, always be busy, limit our exposure to the media, and forget a little about this pandemic, because it can affect our health.

Finally, the present research shows that there is a direct positive significant relationship between stress and anger. On the other hand, significant negative inverse relationships were found that indicated protective factors, such as depression, conscientiousness, and anger, which suggests significant actions for future research. Another limitation of the study was the application of the instrument in a sparsely populated area in a specific region of Peru (Tacna), so it could still be replicated in other regions. In addition, the respondents who were part of the sample were only those who met the desired characteristics and were analyzed as a whole, so a comparison with other subjects separately could be considered. In fact, the sample number was 389, so the sample could be increased to obtain even more robust results.

## Figures and Tables

**Table 1:** Implications of sociodemographic variables on mental health.

KERRYPNX		*N*	%	Stress		Depression		Anxiety	
				Test	*p*-Value	Test	*p*-Value	Test	*p*-Value
Gender	Male	171	44%	−0.847	0.397	−1.647	0.100	−2.996	0.003
	Female	218	56%						
Age Interval	60–79	327	84%	−0.018	0.074	−2.778	0.005	−1.568	0.117
	80 or more	62	16%						
Marital status	Without a partner	160	41%	−0.347	0.729	−0.935	0.350	−0.384	0.701
	With a partner	229	59%						
Children	No children	10	3%	2.823	0.244	1.307	0.520	1.467	0.480
	1–5 children	282	73%						
	≥6 children	97	24%						
Education level	No education	27	7%	9.380	0.052	19.217	0.001	11.602	0.021
	Primary	115	30%						
	Secondary	140	36%						
	Higher	92	24%						
	Exhibit	15	4%						
Residence	Rural	63	16%	−0.633	0.526	−0.467	0.641	−0.272	0.785
	Urban	326	84%						
Cohabitating persons	Lives alone	21	5%	1.518	0.468	1.297	0.523	2.559	0.278
	≤2 people	140	36%						
	≥3 people	228	59%						

**Table 2:** Exposure to COVID-19-related news and association with mental health.

Frequency of Use	No Exposure		Rarely		Sometimes		Frequently		Stress		Depression		Anxiety	
	*N*	%	*N*	%	*N*	%	*N*	%	K-W	*p*-Value	KW	*p*-Value	K-W	*p*-Value
Social Networking	246	63%	73	19%	43	11%	27	7%	2.559	0.465	7.379	0.061	3.325	0.344
Television	66	17%	135	35%	113	29%	75	19%	3.000	0.392	12.646	0.005	2.009	0.570
Radio	54	14%	106	27%	128	33%	101	26%	5.394	0.145	7.432	0.059	5.248	0.154
People	51	13%	148	38%	119	31%	71	18%	5.529	0.137	17.825	0.000	2.329	0.507
Print media	209	54%	107	28%	46	12%	27	7%	3.115	0.374	2.631	0.452	1.711	0.634

**Table 3:** Number of hours of exposure to COVID-19-related news and association with mental health.

Hours of Exposure	No Exposure		1 to 5 h		From 6 h to More		Stress		Depression		Anxiety	
	*N*	%	*N*	%	*N*	%	K-W	*p*-Value	K-W	*p*-Value	K-W	*p*-Value
Social Networking	240	62%	135	35%	14	4%	3.226	0.199	0.016	0.992	5.252	0.072
Television	76	20%	286	74%	27	7%	6.185	0.045	2.271	0.321	1.088	0.580
Radio	70	18%	252	65%	66	17%	5.775	0.056	0.318	0.853	6.346	0.042
People	80	21%	274	70%	35	9%	2.609	0.271	4.255	0.119	1.371	0.504
Print media	238	61%	142	37%	9	2%	0.424	0.809	3.676	0.159	0.292	0.864

**Table 4:** Relationship between psychological consequences and mental health through exposure to news through television, radio, and individuals.

Television	Stress		Depression		Anxiety	
	Spearman’s Rho	*p*-Value	Spearman’s Rho	*p*-Value	Spearman’s Rho	*p*-Value
Anxiety	0.143 **	0.005	0.062	0.225	0.176 **	0.000
Fear	0.101 *	0.047	0.135 **	0.008	0.137 **	0.007
Awareness	−0.002	0.962	−0.175 **	0.001	−0.185 **	0.000
Stress	0.135 **	0.008	0.011	0.827	0.174 **	0.001
Indignation	0.186 **	0.000	−0.133 **	0.009	−0.077	0.127
Security	−0.017	0.735	−0.036	0.480	−0.068	0.179
Nothing	−0.066	0.192	−0.117 *	0.020	−0.112 *	0.027
No use	−0.078	0.124	0.048	0.343	−0.021	0.675
Radio	Spearman’s Rho	*p*-value	Spearman’s Rho	*p*-value	Spearman’s Rho	*p*-value
Anxiety	0.077	0.131	0.063	0.215	0.146 **	0.004
Fear	0.028	0.580	0.173 **	0.001	0.242 **	0.000
Awareness	−0.028	0.585	−0.184 **	0.000	−0.200 **	0.000
Stress	0.151 **	0.003	0.040	0.429	0.135 **	0.008
Indignation	0.118 *	0.020	−0.104 *	0.040	−0.089	0.080
Security	−0.005	0.920	0.016	0.752	0.011	0.827
Nothing	−0.122 *	0.016	−0.041	0.417	−0.003	0.948
No use	−0.021	0.675	0.019	0.710	−0.097	0.057
People	Spearman’s Rho	*p*-value	Spearman’s Rho	*p*-value	Spearman’s Rho	*p*-value
Anxiety	0.010	0.851	0.107 *	0.035	0.189 **	0.000
Fear	0.086	0.089	0.027	0.599	0.155 **	0.002
Awareness	−0.008	0.879	−0.211 **	0.000	−0.148 **	0.003
Stress	0.174 **	0.001	0.041	0.424	0.188 **	0.000
Indignation	0.192 **	0.000	−0.206 **	0.000	−0.071	0.161
Security	−0.031	0.536	0.086	0.089	0.107 *	0.036
Nothing	−0.036	0.478	0.000	0.995	−0.079	0.120
No use	−0.033	0.514	0.137 **	0.007	−0.039	0.448

** Correlation is significant at the 0.01 level (bilateral); * correlation is significant at the 0.05 level (bilateral).

## Data Availability

Data are available upon request from the authors.
